# Supplementary material for: Analysis of the Equilibrium Distribution of Ligands in Heterogeneous Media–Approaches and Pitfalls
Source: Int J Mol Sci. 2022 Aug 28;23(17):9757. doi: 10.3390/ijms23179757 (PMC9478965; doi:10.3390/ijms23179757)
Supplement: Supplementary file 1 [file ijms-23-09757-s001.zip › ijms-1869751-supplementary.pdf]

Supplementary Material for

Analysis of the Equilibrium Distribution of Ligands in Heterogeneous Media – Approaches and Pitfalls

Maria João Moreno,[1,2*] Luís M. S. Loura, [1,3] Jorge Martins,[4] Armindo Salvador,[1,5,6] and Adrian Velazquez-Campoy[7,8,9,10]

[1] Coimbra Chemistry Center—Institute of Molecular Sciences (CQC-IMS), University of Coimbra, 3004-535 Coimbra, Portugal

[2] Department of Chemistry, Faculty of Sciences and Technology, University of Coimbra, 3004-535 Coimbra, Portugal

[3] Faculty of Pharmacy, University of Coimbra, 3000-548 Coimbra, Portugal

[4] Centro de Ciências do Mar (CCMAR/CIMAR, LA), Universidade do Algarve, Campus de Gambelas, 8005-139 Faro, Portugal

[5] CNC—Center for Neuroscience and Cell Biology, 3004-504 Coimbra, Portugal

[6] Institute for Interdisciplinary Research, University of Coimbra, 3030-789 Coimbra, Portugal

[7] Institute for Biocomputation and Physics of Complex Systems (BIFI), Joint Unit GBsC-CSIC-BIFI, Universidad de Zaragoza, 50018 Zaragoza, Spain

[8] Departamento de Bioquímica y Biología Molecular y Celular, Universidad de Zaragoza, 50009 Zaragoza, Spain

[9] Instituto de Investigación Sanitaria de Aragón (IIS Aragon), 50009 Zaragoza, Spain

[10] Centro de Investigación Biomédica en Red en el Área Temática de Enfermedades Hepáticas Digestivas (CIBERehd), 28029 Madrid, Spain

Corresponding author: mmoreno@ci.uc.pt

Index

Section	page
S1 – Additional material for “Proteins with a single binding site”	3
Figure S1 to Figure S3	
S2 – Additional material for “Proteins with more than one binding site”	5
Figure S4 and S5	
S3 – Additional material for “Quenching of Protein Fluorescence - Stern-Volmer plots”	7
Derivation of equations 28 and 29	
Table S1	
Figure S6	

S1 – Additional material for section "Proteins with a single binding site"

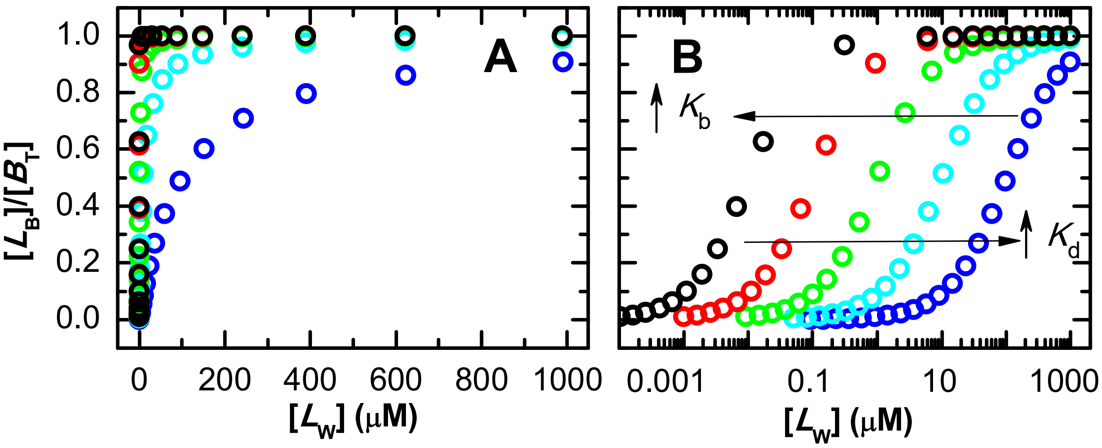

Figure S1 - Saturation of protein binding sites predicted from the full description of the system, (equation (3), as a function of the concentration of free ligand in the aqueous medium, note the logarithm scale on the x axis. The total concentration of binding sites was 10 μM, and the association affinity was varied from K_d equal to 100 μM (○), 10 μM (○), 1 μM (○), 0.1 μM (○), and 0.01 μM (○).

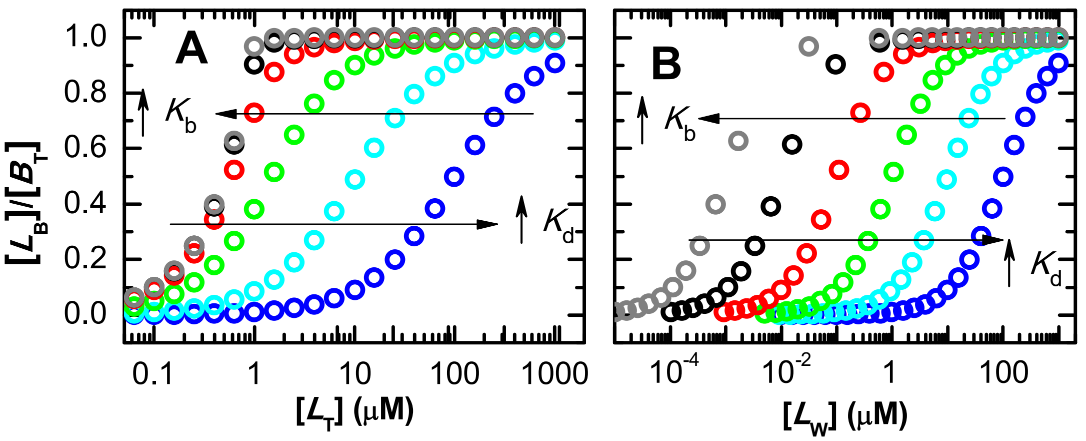

Figure S2 - Saturation of binding sites predicted from the full description of the system, (equation (3), as a function of the concentration of free ligand in the aqueous medium, note the logarithm scale on the x axis. The total concentration of binding sites was 1 μM, and the association affinity was varied from K_d equal to 100 μM (○), 10 μM (○), 1 μM (○), 0.1 μM (○), 0.01 μM (○), and 0.001 μM (○).

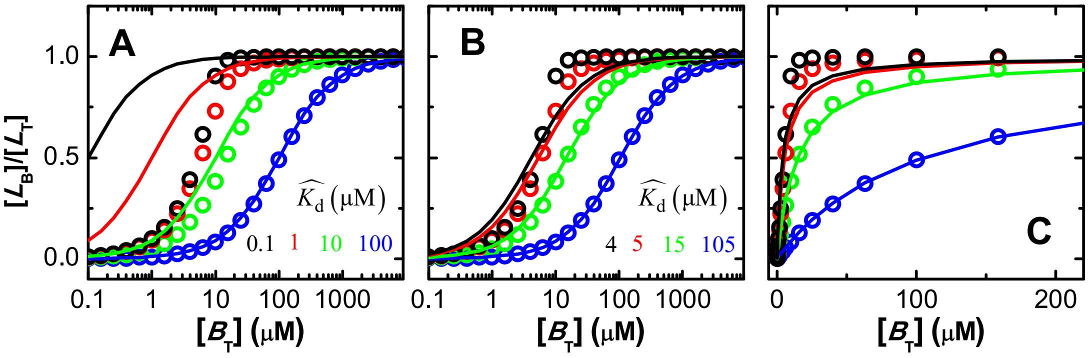

Figure S3 – Fraction of bound ligand predicted from the full description of the system (equation (3), circles) for $[L_T]$ = 10 μM and different binding affinities: K_d =100 μM (○), 10 μM (○), 1 μM (○), and 0.1 μM (○). The lines where calculated from equation (6) (excess ligand approximation). Plot A – fraction of ligand bound predicted for the "true" binding affinities; Plots B and C – best fit of equation (6) to the saturation of binding sites observed (note the logarithmic scale in plot B and linear scale in plot C). The dissociation constants obtained from the best fit of equation (6) are indicated in plot B.

S2 – Additional material for section “Proteins with more than one binding site”

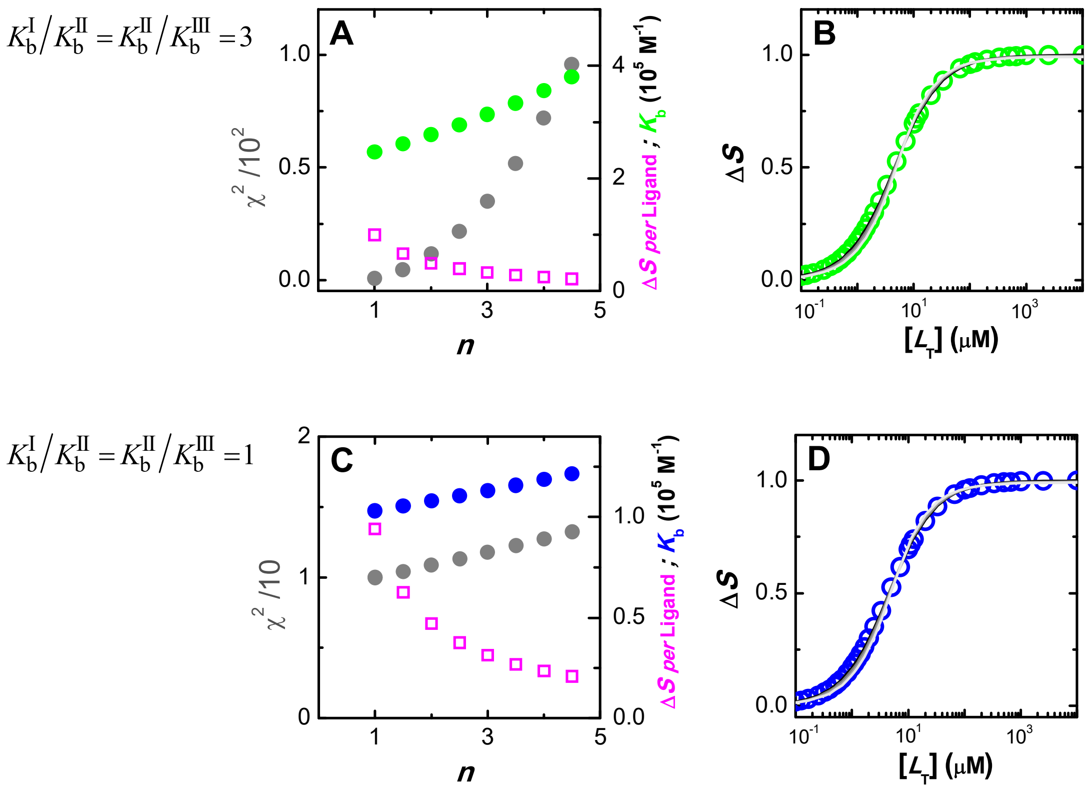

Figure S4 – Sensitivity to the number of binding sites (n) and average binding affinity (K_b) when the protein saturation profile obtained for the case of different but independent binding sites is analysed with the assumption that the binding sites are all equal and independent, equation (3), for the case of $K_b^{I}/K_b^{II} = K_b^{II}/K_b^{III} = 3$ (plots A and B) and $K_b^{I}/K_b^{II} = K_b^{II}/K_b^{III} = 10$ (plots C and D). The effect of the number of binding sites *per* protein in the quality of the best fit (●), association constant (● or ●) and signal variation per ligand bound (□), is shown in plots A and C. The variation in the signal from the protein (○ or ○) and the best fit curves for n=1 (—), n=2 (—), n=3 (—), and n=4 (—) are shown in plots B and D.

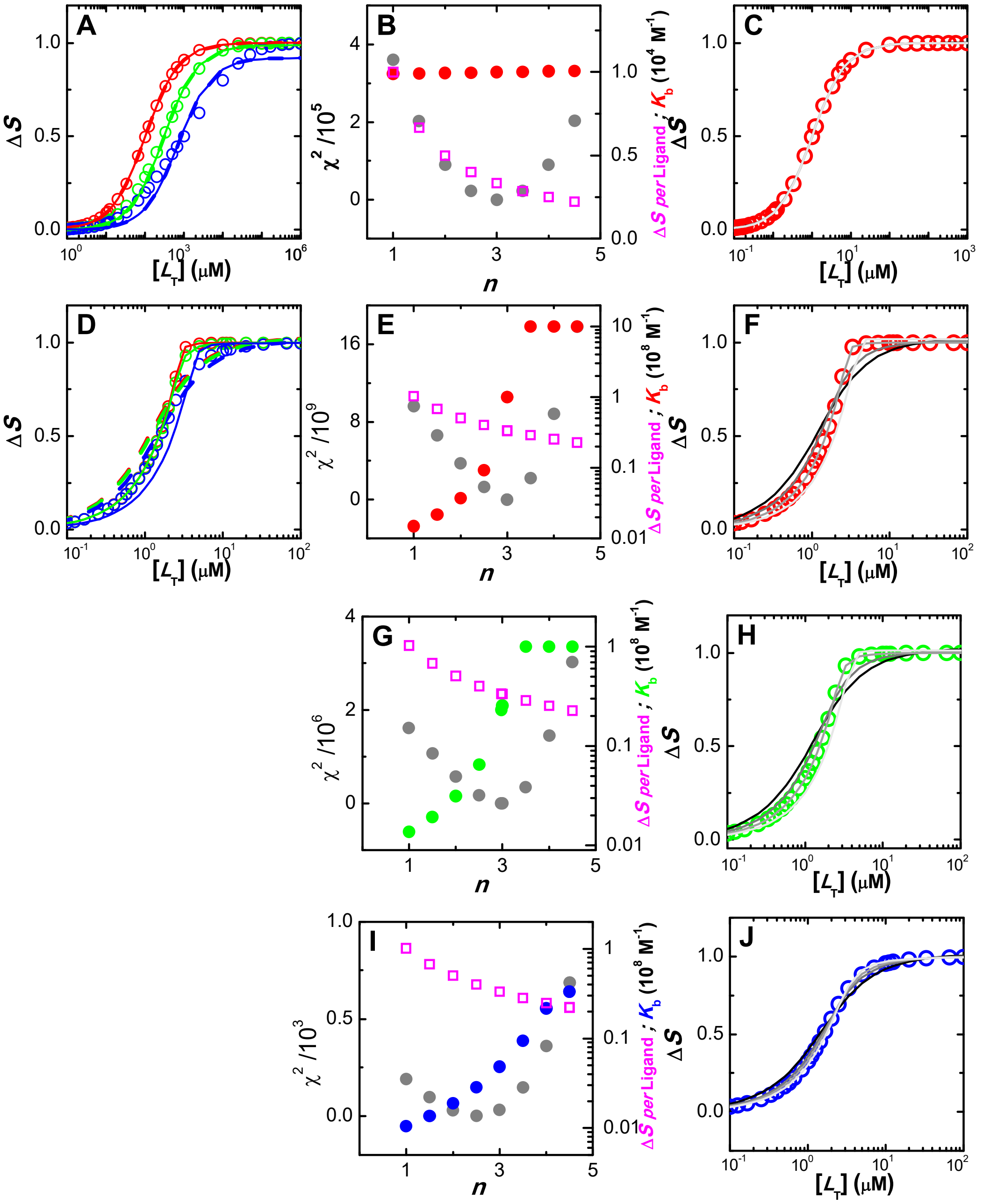

Figure S5 – Plots A to C: variation of a hypothetical signal proportional to the number of ligands associated with a protein containing 3 independent binding sites (K_b^{I}=10^4 M^{-1}; and $K_b^{I}/K_b^{II} = K_b^{II}/K_b^{III} = 10$ ○, $K_b^{I}/K_b^{II} = K_b^{II}/K_b^{III} = 3$ ○, and $K_b^{I}/K_b^{II} = K_b^{II}/K_b^{III} = 1$ ○. The lines are the best fit considering only one type of binding sites, equation (3), with the number of binding sites fixed at 1 (thick dashed lines) or as an adjustable variable (thin continuous lines) (plot A). Effect of the number of binding sites *per* protein in the quality of the best fit (●), association constant (●) and signal variation per ligand bound (□), for the case $K_b^{I}/K_b^{II} = K_b^{II}/K_b^{III} = 1$ (plot B), and representation of the signal variation for the fitting curves obtained for n=1 (—), n=2 (—), n=3 (—), and n=4 (—) (plot C). Plot D to F: Equivalent to plots A to C but for K_b^{I}=10^8 M^{-1}. The corresponding to plots E and F are represented in plots G and H, and I and J, for the case of $K_b^{I}/K_b^{II} = K_b^{II}/K_b^{III} = 3$ ○, and $K_b^{I}/K_b^{II} = K_b^{II}/K_b^{III} = 1$ ○, respectively.

S3 – Additional material for section "Quenching of Protein Fluorescence - Stern-Volmer plots"

Derivation of equations 28 and 29

The master equation for the variation of the signal from the protein in the presence of ligand, S, is given below (derived from equation (17) in the main manuscript):

$$S = s_0[P] + \sum_{i=1}^{N} s_\mathrm{i}\left[PL^\mathrm{i}\right],$$

leading to the following equation for S_0/S :

$$\frac{S_0}{S} = \frac{s_0[P_\mathrm{T}]}{s_0[P] + \sum_{i=1}^{N} s_\mathrm{i}\left[PL^\mathrm{i}\right]},$$

For low concentrations of ligand, it may be assumed that the proteins have only one bound ligand. Depending on the relative binding affinities for the distinct binding sites, the ligand may be always in one binding site (that with the highest binding affinity) or distributed among sites with similar binding affinities. In any case, it may be assumed that the concentration of proteins with more than one binding site is negligible, and the above equation simplifies to:

$$\frac{S_0}{S} = \frac{s_0[P_\mathrm{T}]}{s_0[P] + \sum_{i=1}^{n} s_\mathrm{i}\left[PL_1^\mathrm{i}\right]},$$

where s_i is the residual fluorescence of the protein when it has only one ligand molecule that is in site i, and $\left[PL_1^\mathrm{i}\right]$ is the concentration of this set of proteins. The variable $\left[PL_1^\mathrm{i}\right]$ depends on the binding affinity for the binding site, K_b^i, and on the concentrations of free ligand and protein:

$$\left[PL_1^\mathrm{i}\right] = K_\mathrm{b}^\mathrm{i}[P][L_\mathrm{W}].$$

The equation for S_0/S becomes:

$$\frac{S_0}{S} = \frac{s_0[P_\mathrm{T}]}{s_0[P] + \sum_{i=1}^{n} s_\mathrm{i} K_\mathrm{b}^\mathrm{i}[P][L_\mathrm{W}]}$$

Replacing S_0/S by its relation with K_SV, and the total concentration of protein by the sum of all relevant species at low saturation leads to:

$$1 + K_\mathrm{SV}[L_\mathrm{W}] = \frac{s_0\left([P] + \sum_{i=1}^{n} K_\mathrm{b}^\mathrm{i}[P][L_\mathrm{W}]\right)}{s_0[P] + \sum_{i=1}^{n} s_\mathrm{i} K_\mathrm{b}^\mathrm{i}[P][L_\mathrm{W}]} = \frac{1 + \sum_{i=1}^{n} K_\mathrm{b}^\mathrm{i}[L_\mathrm{W}]}{1 + \sum_{i=1}^{n} \frac{s_\mathrm{i}}{s_0} K_\mathrm{b}^\mathrm{i}[L_\mathrm{W}]},$$

and multiplying the left and the right expressions leads to:

$$1+\sum_{i=1}^{n}\frac{s_\text{i}}{s_0}K_\text{b}^\text{i}\left[L_\text{W}\right]+K_\text{SV}\left[L_\text{W}\right]+K_\text{SV}\sum_{i=1}^{n}\frac{s_\text{i}}{s_0}K_\text{b}^\text{i}\left[L_\text{W}\right]^2=1+\sum_{i=1}^{n}K_\text{b}^\text{i}\left[L_\text{W}\right]$$

$$K_\text{SV}\left(\left[L_\text{W}\right]+\sum_{i=1}^{n}\frac{s_\text{i}}{s_0}K_\text{b}^\text{i}\left[L_\text{W}\right]^2\right)+\sum_{i=1}^{n}\frac{s_\text{i}}{s_0}K_\text{b}^\text{i}\left[L_\text{W}\right]-\sum_{i=1}^{n}K_\text{b}^\text{i}\left[L_\text{W}\right]=0 .$$

If the ligand concentration is low enough, the terms in $\left[L_\text{W}\right]^2$ may be neglected $\left(\left[L_\text{W}\right]\ll 1\Big/\sum_{i=1}^{n}\frac{s_\text{i}}{s_0}K_\text{b}^\text{i}\right)$, leading to equation (29) of the main manuscript.

$$K_\text{SV}\left[L_\text{W}\right]=\sum_{i=1}^{n}K_\text{b}^\text{i}\left[L_\text{W}\right]-\sum_{i=1}^{n}\frac{s_\text{i}}{s_0}K_\text{b}^\text{i}\left[L_\text{W}\right]$$

$$K_\text{SV}\left[L_\text{W}\right]=\sum_{i=1}^{n}\left(1-\frac{s_\text{i}}{s_0}\right)K_\text{b}^\text{i}\left[L_\text{W}\right]$$

$$K_\text{SV}=\sum_{i=1}^{n}\left(1-\frac{s_\text{i}}{s_0}\right)K_\text{b}^\text{i}$$

Equation (28) is obtained from the above equation by assuming that s_i = 0 for all i .

Table S1 – Parameters obtained from the best fit of the simulated results (for proteins with 3 independent binding sites) with the different procedures indicated in the manuscript. The values indicated for χ^2 are relative to those obtained for the different n in the set for the same fitting method (small S_0/S or whole variation). The estimates within 10 % of the true overall binding affinity are shaded in green, those within 30 % are shaded blue, in the same order of magnitude are shaded yellow, and estimates deviated by more than an order of magnitude are shaded in red.

Simulated ("true") parameters								Parameters obtained from the best fit at small S_0/S (assuming $s_\mathrm{i}=0$)						Parameters obtained from the best fit of the whole S_0/S variation (up to 10)								
								$n=1$		$n=2$		$n=3$		$n=1$			$n=2$			$n=3$		
$[P_\mathrm{T}]$ (10 $^{-6}$ M)	K_b^I (M)	K_b^II (M)	$K_\mathrm{b}^\mathrm{III}$ (M)	$\frac{s_\mathrm{I}}{s_0}$	$\frac{s_\mathrm{I}}{s_0}$	$\frac{s_\mathrm{I}}{s_0}$	max $\frac{S_0}{S}$	$\frac{n K_\mathrm{b}}{\sum K_\mathrm{b}^\mathrm{i}}$	χ^2	$\frac{n K_\mathrm{b}}{\sum K_\mathrm{b}^\mathrm{i}}$	χ^2	$\frac{n K_\mathrm{b}}{\sum K_\mathrm{b}^\mathrm{i}}$	χ^2	$\frac{n K_\mathrm{b}}{\sum K_\mathrm{b}^\mathrm{i}}$	$\frac{s_\mathrm{i}}{s_0}$	χ^2	$\frac{n K_\mathrm{b}}{\sum K_\mathrm{b}^\mathrm{i}}$	$\frac{s_\mathrm{i}}{s_0}$	χ^2	$\frac{n K_\mathrm{b}}{\sum K_\mathrm{b}^\mathrm{i}}$	$\frac{s_\mathrm{i}}{s_0}$	χ^2
1		10^4			0		10	1.0	1.0	1.0	1.1	1.0	1.1	2.0	0	10^{11}	1.2	0	10^{10}	1.0	0	1.0
				0.6	0.3	0	5	0.7	1.0	0.7	1.1	0.7	1.1	0.93	0	10^3	0.7	0.04	1.0	0.7	0.08	21
				1	1	0	2.3	0.33	1.0	0.33	1.0	0.33	1.0	0.33	0	1.0	0.37	0.16	4.8	0.4	0.21	11
		10^6			0		10	0.97	1.0	1.1	1.9	1.1	5.0	1.27	0	10^6	1.0	0	10^4	1.0	0	1.0
				0.6	0.3	0	10	0.37	1.0	0.37	30	0.40	50	0.73	0	10^2	0.43	0	4	0.43	0.02	1.0
				1	1	0	10	0.11	1.0	0.11	1.6	0.11	1.8	0.27	0	46	0.17	0.03	2	0.16	0.05	1.0
1		10^8			0		10	0.1	1.0	1.0	2.7	33	8.9	0.04	0	10^9	0.06	0	10^6	1.0	0	1.0
				0.6	0.3	0	8	0.01	1.0	0.01	10^2	0.01	10^3	0.02	0	4.5	0.01	0	1.2	0.02	0	1.0
				1	1	0	3	0.002	1.6	0.002	1.0	0.002	2.8	0.004	0	1.6	0.003	0	1.1	0.003	0	1.0
0.1				0.6	0.3	0	10	0.07	1.0	0.08	10^2	0.08	10^3	0.12	0	5.4	0.11	0	1.3	0.12	0	1.0
0.01				0.6	0.3	0	10	0.4	1.0	0.4	24	0.4	40	0.72	0	10^2	0.43	0	4.1	0.43	0.02	1.0

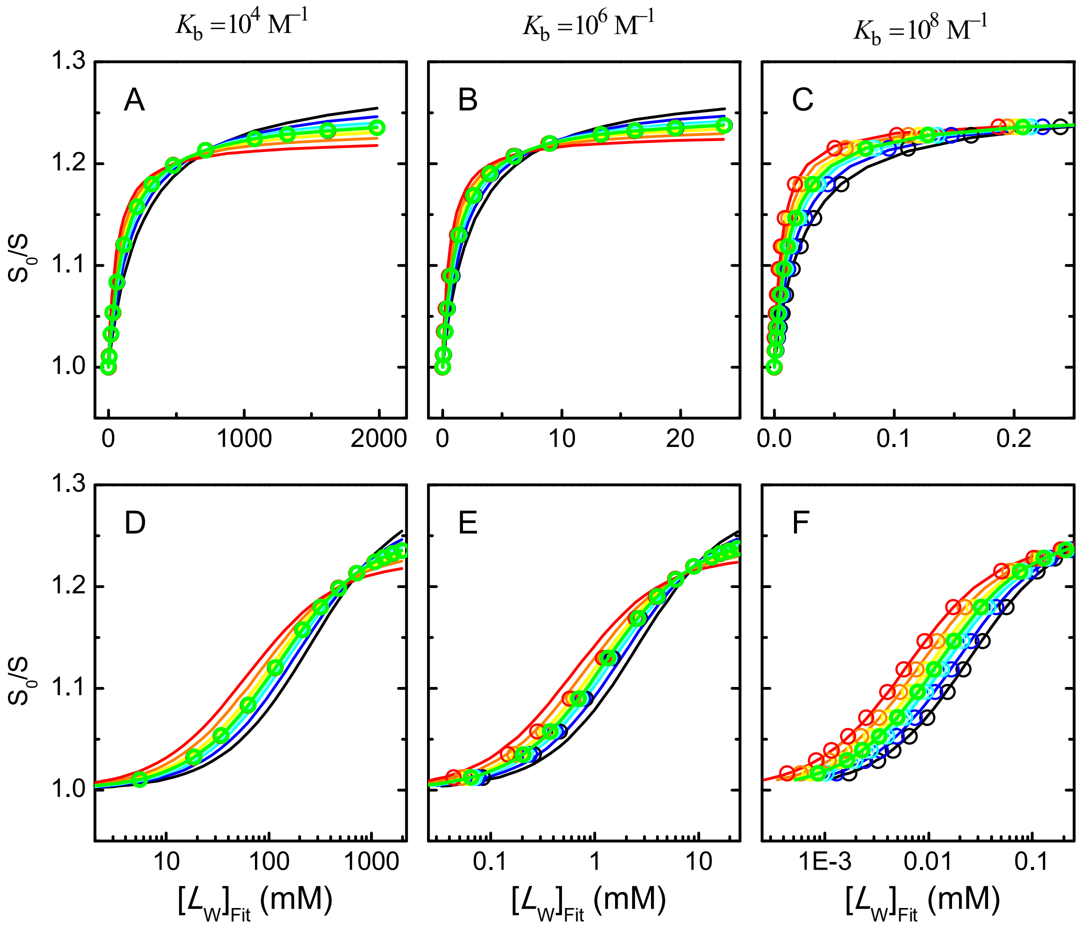

Figure S6 – Sensitivity of the best fit of equation **Error! Reference source not found.** to the whole variation of S_0/S for the case of low (left), intermediate (middle) and high (right) binding affinity, and $[P_T] = 10^{-6}\,\mathrm{M}$. The variation of S_0/S with $[L_W]_{Fit}$ calculated from the "true" parameters is shown in green (○, and —), and that corresponding to different ratios between the estimated and the true K_b is shown by different colours: estimated/true= 0.5 (○, and —), 0.67 (○, and —), 0.83 (○, and —), 0.91 (○, and —), 1.1 (○, and —), 1.2 (○, and —), 1.5 (○, and —), and 2 (○, and —). Note the logarithmic scale for the x axis in the lower plots.
